# Health utility, work productivity, and total economic burden of polycystic ovary syndrome

**DOI:** 10.1210/jendso/bvag217

**Published:** 2026-09-28

**Authors:** Mina Amiri, Yucheng Huang, Linda Thai, Sasha Ottey, William Patterson, Olivia R Delau, Surabhi Yadav, Kendall Soucie, Ty Partridge, Richard P Buyalos, Ricardo Azziz

**Affiliations:** Maternal, Fetal, & Neonatal Research Center, Family Health Institute,Tehran University of Medical Sciences (TUMS), Tehran 1419733141, Iran; Foundation for Research and Education Excellence, Vestavia, AL 35243, USA; PCOS Challenge: The National Polycystic Ovary Syndrome Association, Atlanta, GA 30308, USA; PCOS Challenge: The National Polycystic Ovary Syndrome Association, Atlanta, GA 30308, USA; PCOS Challenge: The National Polycystic Ovary Syndrome Association, Atlanta, GA 30308, USA; PCOS Challenge: The National Polycystic Ovary Syndrome Association, Atlanta, GA 30308, USA; PCOS Challenge: The National Polycystic Ovary Syndrome Association, Atlanta, GA 30308, USA; NYU School of Global Public Health, New York University, New York, NY 10003, USA; PCOS Challenge: The National Polycystic Ovary Syndrome Association, Atlanta, GA 30308, USA; NYU School of Global Public Health, New York University, New York, NY 10003, USA; Department of Psychology, University of Windsor, Windsor, ON N9B 3P4, Canada; Department of Psychology, Wayne State University, Detroit, MI 48202, USA; Foundation for Research and Education Excellence, Vestavia, AL 35243, USA; Department of Obstetrics & Gynecology, Heersink School of Medicine, University of Alabama at Birmingham (UAB), Birmingham, AL 35233, USA; Foundation for Research and Education Excellence, Vestavia, AL 35243, USA; Department of Obstetrics & Gynecology, Heersink School of Medicine, University of Alabama at Birmingham (UAB), Birmingham, AL 35233, USA; Department of Medicine, Heersink School of Medicine, UAB, Birmingham, AL 35233, USA; Department of Healthcare Organization & Policy, School of Public Health, UAB, Birmingham, AL 35233, USA

**Keywords:** polycystic ovary syndrome, polyendocrine metabolic ovarian syndrome, quality-adjusted life years, work productivity, cost of illness, health utility

## Abstract

**Background:**

Polycystic ovary syndrome (PCOS), also known as polyendocrine metabolic ovarian syndrome (PMOS), impairs health-related quality of life (QoL) and daily functioning. While estimates of medical-related costs have been made, this study aimed to estimate indirect (work productivity-related) and intangible (QoL-related) economic costs.

**Methods:**

In a cross-sectional US registry (PCOS/PMOS = 433, controls = 252), we measured willingness to pay, health utility using the EuroQol five-dimension five-level (EQ-5D-5L) and the Short Form 6-Dimensions (SF-6D) tools, and work productivity with Work Productivity and Activity Impairment: General Health. Adjusted regression estimated utility differences were converted to quality-adjusted life year (QALY) loss. XGBoost classification with Shapley Additive exPlanations feature importance identified key discriminating domains.

**Results:**

PCOS/PMOS had lower health utility compared to controls (EQ-5D-5L, 0.72 vs 0.88; SF-6D, 0.67 vs 0.72; *P* < .001), corresponding to QALY losses of 0.12 and 0.04, respectively. Annual intangible costs per woman were then estimated to be $12 000 (per EQ-5D-5L) or $4000 (per SF-6D). Median work productivity loss for PCOS/PMOS was 39.5% vs 6.0% for controls (using $37/hour and 2080 hours/year). Considering the number of women with PCOS/PMOS in the United States, the total yearly indirect cost was estimated to be $97.9 billion, and yearly intangible cost was estimated to range from $17.8 billion (by SF-6D) to $53.4 billion (by EQ-5D-5L), in 2026 USD. Machine learning identified pain, anxiety/depression, and physical functioning limitations as key discriminators.

**Conclusion:**

PCOS/PMOS is associated with lower utility, significant QALY losses, and work productivity impairment. Considering prior estimates of medical-related costs, the total annual US Societal burden ranges from $133 to $169 billion, predominantly from indirect and intangible costs, underscoring the profound functional and economic impact of the disorder.

Polycystic ovary syndrome (PCOS), also known as polyendocrine metabolic ovarian syndrome (PMOS), is the most common endocrine disorder among women of reproductive age, affecting approximately 6% to 11% of women globally (1), with an increasing burden over recent decades (2). It is characterized by menstrual dysfunction, hyperandrogenism, and polycystic ovary morphology (3) and is recognized as a multisystem condition extending beyond reproductive dysfunction. PCOS/PMOS is associated with cardiometabolic risks (3-8), adverse pregnancy outcomes (9), gynecological malignancies (10, 11), psychological distress (12-17), sleep disturbances (18), and impairments in daily functioning that affect social and occupational roles (19, 20). Given this multisystem nature, an international consensus process recently renamed the condition polyendocrine metabolic ovarian syndrome (PCOS/PMOS) (21). Moreover, symptoms and comorbidities associated with PCOS/PMOS, including pain, menstrual disturbances, fatigue, sleep disruption, psychological distress, metabolic complications, and fertility-related concerns, may affect daily functioning, work attendance, and on-the-job productivity (22-26), highlighting the importance of examining work-related outcomes in PCOS/PMOS.

Women with PCOS/PMOS consistently report poorer health-related quality of life (QoL) (12, 15), driven by both clinical manifestations, such as hirsutism, acne, weight concerns, and infertility (12, 27, 28), and long-term comorbidities, including type 2 diabetes mellitus (T2DM) (16, 29) and depression (30). Generic instruments, such as the EuroQol five-dimension five-level (EQ-5D-5L) and the 36-item Short Form (SF-36), are widely used to measure the multidimensional impact of chronic conditions and to derive preference-based health utility scores (15-17, 27, 29, 31-33). Health utility values, which reflect the desirability of a health state, provide standardized metric for comparing disease burden across conditions and are central to economic evaluation (33-36). However, preference-based utility estimates that directly compare women with and without PCOS/PMOS remain scarce.

PCOS/PMOS also imposes a substantial economic and societal burden. Annual direct healthcare costs in the United States exceed $15 billion, with major contributions from mental health disorders, pregnancy complications, and cardiometabolic outcomes (13). The total economic burden of a disease, however, is composed of 3 elements: direct healthcare expenditures, indirect costs (productivity losses), and intangible costs (the monetary value of pain, suffering, and reduced QoL). Indirect costs, including work absenteeism, presenteeism, and activity impairment, may also be substantial (19). In addition to these measurable costs, intangible costs, reflected in diminished well-being and reduced QoL, can be captured through preference-based health utility measures (13). Symptoms such as menstrual irregularities, pain, fatigue, mood disturbances, and sleep disorders, together with obesity and metabolic comorbidities, likely impair work attendance and on-the-job performance (14, 18, 37, 38). However, quantitative evidence on work productivity loss associated with PCOS/PMOS remains limited.

Although previous studies have quantified the direct healthcare expenditures attributable to specific PCOS-/PMOS-related conditions, including reproductive care, pregnancy complications, T2DM, stroke, mental health disorders, and endometrial cancer (10, 13, 39, 40), no study has simultaneously measured the intangible and indirect costs of PCOS/PMOS in a single controlled comparison. Consequently, the full economic burden—the sum of direct, indirect, and intangible costs—has never been comprehensively estimated. Closing this gap is critical for accurately appraising the societal impact of PCOS/PMOS and for guiding healthcare policy and resource allocation.

The present study among US women therefore aimed to (1) compare QoL, health utility, and work productivity between women with and without PCOS/PMOS; (2) estimate the independent association of PCOS/PMOS with health utility using multivariable and quantile regression; (3) calculate the excess quality-adjusted life year (QALY) loss and corresponding intangible monetary burden relative to controls from both the EQ-5D-5L and the Short Form 6-Dimensions (SF-6D) questionnaires; (4) quantify the indirect cost attributable to PCOS/PMOS from the observed work productivity impairment; (5) combine these intangible and indirect cost estimates with existing direct healthcare cost data to determine, for the first time, the total annual economic burden of PCOS/PMOS in the United States; and (6) use machine learning to identify which health domains most strongly differentiate women with PCOS/PMOS from controls.

## Materials and methods

### Study design and data collection

This cross-sectional study analyzed data from the PCOS Challenge Study patient registry (protocol PCOSC001), a US health registry of women with and without PCOS/PMOS. The study was approved by the Genetic Alliance Institutional Review Board (IRB). Data were collected through secure online surveys administered in modular format. Participants initially completed registry surveys on the LunaDNA platform; after LunaDNA ceased operations in 2024, identical survey instruments were migrated to Qualtrics under an IRB-approved amendment (May 9, 2024). The transition preserved instrument wording, scoring, branching logic, and response options to ensure measurement continuity across platforms. All participants provided electronic informed consent prior to completing the online questionnaire.

### Participants and recruitment

This study enrolled adult women (≥18 years) residing in the United States who were proficient in English and capable of completing web-based questionnaires. Recruitment followed a nonprobability sampling design. Women with PCOS/PMOS were initially identified through the LunaDNA platform between September 2022 and May 2023; participants reported either a physician-confirmed diagnosis of PCOS/PMOS or self-identified as having the condition. Among 433 participants with PCOS/PMOS, diagnosis source was available for 432; 1 participant was missing this information. Of these, 98.1% (n = 424) reported being physician diagnosed and 1.9% (n = 8) self-identified. After transitioning to Qualtrics, recruitment of additional PCOS/PMOS participants and all control participants proceeded between May 2024 and August 2024. Control participants were recruited via a snowball sampling method, wherein participants with PCOS/PMOS were invited to refer acquaintances without a history of PCOS/PMOS. To minimize misclassification, control participants were required to report no prior PCOS/PMOS diagnosis and screen negative on a structured symptom questionnaire covering hirsutism, menstrual irregularity, hair thinning, moderate to severe acne, and excess weight gain. Although clinical examination was not feasible, this conservative symptom-based screening approach minimized the likelihood of including women with undiagnosed PCOS/PMOS. Participants contributed to analyses only if they completed the relevant survey instrument; therefore, analytic sample sizes vary across outcomes. No formal a priori sample size calculation was performed; the sample size was determined by recruitment feasibility during the study period.

### Measures

Participants completed standardized questionnaires capturing demographic and clinical characteristics, including age, race, ethnicity, education levels, income, employment status, marital status, sexual orientation, and anthropometric measures used to calculate body mass index (BMI). Health-related QoL was measured using the EQ-5D-5L and the 36-Item Short Form Survey (SF-36v1). The EQ-5D-5L assesses 5 dimensions of health status—mobility, self-care, usual activities, pain/discomfort, and anxiety/depression—and includes a self-rated visual analog scale (ie, EQ-VAS) ranging from 0 (worst imaginable health) to 100 (best imaginable health) (41). The SF-36 measures 8 domains of physical and mental health, using standard scoring procedures (each domain scored 0-100, higher scores indicating better health) (32).

Preference-based health utility scores were derived as follows: The EQ-5D-5L utility index was generated using the standard US value set (42). The SF-6D utility score was obtained from SF-36 responses using the Brazier algorithm, which applies UK population preference weights (33, 36). Both utility indices serve as the quality-of-life weight for subsequent QALY estimation.

Work productivity and activity impairment were assessed using the Work Productivity and Activity Impairment: General Health (WPAI-GH) questionnaire, which evaluates absenteeism, presenteeism, overall work productivity loss, and activity impairment over the preceding 7 days (43). Participants with PCOS/PMOS also completed an open-ended contingent valuation question indicating the maximum amount they would be willingness to pay (WTP), within their financial means, for a complete cure for PCOS/PMOS without adverse effects.

### Definitions

PCOS/PMOS status was self-reported as either physician diagnosed or self-identified. Although formal diagnostic criteria were not independently verified, participants reported symptom profiles consistent with core clinical features of PCOS/PMOS. Quality of life, health utility, and productivity outcomes were calculated according to established scoring algorithms for each instrument.

### Outcomes

The primary outcomes were health utility values (EQ-5D-5L index, SF-6D) and work productivity and activity impairment (WPAI-GH: absenteeism, presenteeism, overall work productivity loss, activity impairment). Secondary outcomes included EQ-5D-5L dimension-level problems, EQ-VAS scores, and SF-36 domain scores. Derived economic outcomes comprised the annual QALY loss and its monetized intangible cost, the indirect (productivity) excess cost, and the total annual economic burden (direct plus indirect plus intangible costs). Machine-learning feature importance (key discriminating health domains) was an ancillary analytic outcome. Willingness to pay (WTP) for a complete cure was an exploratory patient-centric outcome.

### Monetization of intangible (quality of life-related) costs: quality-adjusted life years estimation

In a cross-sectional study, the utility score at a single time point is assumed to represent the individual's typical health over 1 year, so the score directly equals the annual QALYs. The annual QALY loss attributable to PCOS/PMOS was taken as the absolute value of the adjusted mean utility difference (the ordinary least squares [OLS] regression coefficient for PCOS/PMOS status) from models adjusting for age, BMI, and sociodemographic factors (34, 44). This loss was then monetized using societal WTP thresholds: $100 000 per QALY as the base case, with $50 000 and $150 000 in sensitivity analyses (45). Annual monetary loss = QALY loss × WTP threshold. Confidence intervals were derived by multiplying the 95% CI limits of the QALY loss by the threshold. Self-reported WTP for a cure is reported descriptively for the PCOS/PMOS group only.

### Monetization of indirect (work productivity-related) costs

Indirect costs were calculated using the human capital method (43, 46): annual indirect cost per employed woman = (overall work productivity loss % ÷ 100) × hourly wage × annual work hours. We used the US average private-sector hourly wage of $37.00 (May 2026) (47), which is consistent with the income distribution of our study sample (Table 1), where the median annual household income category was $50 000 to $99 999, corresponding to an approximate hourly wage of $24 to $48 for a single full-time earner, and 2080 work hours per year (40 hours/week × 52 weeks), a standard assumption in productivity costing. Because WPAI-GH data exist only for employed women, each group's per-employed cost was multiplied by the employment rate (PCOS/PMOS, 84.53%; controls, 79.37%; from Table 1) to obtain the per-woman cost (including nonemployed). The indirect excess cost is the difference between the PCOS/PMOS and control per-woman costs. All formulas and sources are detailed in Table S1 (48).

**Table 1:** Baseline demographic and clinical characteristics of women with and without PCOS/PMOS

Variables	PCOS/PMOS (N = 433)	Non-PCOS/PMOS controls (N = 252)	*P* value	SMD
Age (years)				
18-25	34 (7.85)	50 (19.84)	<.001	−0.312
26-35	245 (56.58)	67 (26.59)		
36-45	128 (29.56)	71 (28.17)		
46-64	24 (5.54)	51 (20.24)		
≥65	2 (0.46)	13 (5.16)		
Race/ethnicity				
White or Caucasian	318 (73.44)	151 (59.92)	<.001	−0.120
Black or African American	52 (12.01)	50 (19.84)		
Native American or American Indian	6 (1.39)	0 (0.00)		
Asian	4 (0.92)	36 (14.29)		
Two or more races	33 (7.62)	9 (3.57)		
Other	18 (4.16)	5 (1.98)		
Prefer not to say	2 (0.46)	1 (0.40)		
Sexual orientation				
Straight/heterosexual	344 (79.45)	217 (86.11)	.015	0.177
Nonheterosexual	89 (20.55)	35 (13.89)		
Education level				
High school or less	40 (9.24)	3 (1.19)	<.001	−0.317
Bachelor's degree or equivalent	253 (58.43)	143 (56.75)		
Master's degree or higher level	140 (32.33)	106 (42.06)		
Employment status				
Currently employed	366 (84.53)	200 (79.37)	.143	−0.134
Unemployed	67 (15.47)	52 (20.63)		
AHI (USD)				
Less than $10 000	15 (3.61)	10 (3.97)	<.001	−0.469
$10 000 to $49 999	113 (27.23)	39 (15.48)		
$50 000 to $99 999	176 (42.17)	81 (32.14)		
$100 000 to $149 999	76 (18.31)	56 (22.22)		
≥$150 000	36 (8.67)	66 (26.10)		
Prefer not to say	18 (4.16)	0 (0.00)		
Health insurance type				
Government/national/social insurance	15 (3.46)	14 (5.56)	<.001	0.220
Private health insurance	28 (6.47)	44 (17.46)		
Employer-sponsored insurance	289 (66.74)	153 (60.71)		
Medicare	2 (0.46)	8 (3.17)		
Medicaid or government assistance plan	43 (9.93)	8 (3.17)		
TRICARE or other military health care	4 (0.92)	5 (1.98)		
Veterans Affairs (VA)	0 (0.00)	1 (0.40)		
Indian Health Service (for Native Americans and Alaska Natives)	2 (0.46)	0 (0.00)		
Other	6 (1.39)	2 (0.79)		
Multiple insurance plans	31 (7.16)	14 (5.56)		
No health insurance	13 (3.00)	3 (1.19)		
Marital status				
Married/domestic partnership	269 (62.12)	121 (48.02)	<.001	−0.286
Single/divorced/widowed/other	165 (37.88)	131 (51.98)		
PCOS/PMOS duration				
<1 year	20 (4.62)	—		
1 to 5 years	114 (26.33)	—		
6 to 10 years	99 (22.87)	—		
11 to 15 years	85 (19.63)	—		
16 to 20 years	74 (17.09)	—		
≥21 years	32 (7.39)	—		
Missing	9 (2.08)	—		
Height (inches)				
Mean ± SD	64.73 ± 2.74	64.37 ± 3.42	.0557	0.116
Weight (pounds)				
Median (IQR)	225 (190, 270)	152 (130, 187.5)	<.001	1.228
BMI, kg/m^2^				
Median (IQR)	38.35 (31.75, 44.59)	25.77 (22.59, 31.90)	<.001	1.257
BMI category				
Underweight	2 (0.46)	5 (1.98)	<.001	1.240
Healthy weight	24 (5.54)	104 (41.27)		
Overweight	54 (12.47)	70 (27.78)		
Obesity	353 (81.52)	73 (28.97)		

Values are n (%), mean ± SD, or median (IQR).

Abbreviations: AHI, annual household income; BMI, body mass index; SMD, standardized mean difference.

### National aggregate calculation of costs

To estimate the national aggregate burden, the per-woman indirect and intangible costs derived from the present study were combined with previously published direct healthcare cost estimates, which were not derived from our registry sample. These published estimates encompass mutually exclusive cost categories and were adjusted to 2026 USD using the Medical Care Consumer Price Index (49); full derivation and sources are provided in Table S1 (48). The US female population aged 15-44 years is 67 402 866 (50). Considering a 6.6% prevalence (1) of classic PCOS/PMOS (phenotypes A and B) PCOS/PMOS (51, 52), clinical presentations that are the most closely associated with metabolic morbidity (53-55), yields a total of 4 448 589 affected women currently in the United States. Per-woman excess costs were multiplied by the number of affected women in the United States, and all aggregate costs were rounded to the nearest $0.1 billion.

### Statistical analysis

Continuous variables were summarized as means ± SD or medians (IQR), and categorical variables as frequencies/percentages. Between-group comparisons used χ^2^ and Mann–Whitney U tests; standardized mean differences (SMDs) quantified effect sizes. Multivariable associations of PCOS/PMOS with utility were estimated by OLS regression with robust standard errors, adjusting for age, BMI category, race, sexual orientation, education, employment, income, and marital status. Age group was included as a covariate to account for the observed age differences between the PCOS/PMOS and control groups, as design-based age matching was not employed. Quantile regression assessed heterogeneity across the utility distribution. Machine-learning classification was performed using the XGBoost algorithm. Separate models were trained with (1) EQ-5D-5L dimension scores and EQ-VAS, (2) SF-36 domain scores, and (3) WPAI-GH metrics as predictors and PCOS/PMOS as the binary outcome. Feature importance was identified using SHAP (Shapley Additive exPlanations) values to identify the domains most strongly associated with discriminating PCOS/PMOS from controls. Missing data were minimal (<5%), and complete-case analysis was used. All analyses used SPSS 29, Stata 18, and R 4.4.2, following STROBE and CHEERS guidelines.

## Results

### Participant characteristics

A total of 685 women were included (PCOS/PMOS n = 433; controls n = 252). Women with PCOS/PMOS were younger, had higher BMI, and were more likely to be White, married/partnered, and nonheterosexual. Employment rates were 84.53% (PCOS/PMOS) and 79.37% (controls). All characteristics are fully detailed in Table 1.

### Health-related utility and quality of life

Women with PCOS/PMOS reported significantly more problems across all EQ-5D-5L dimensions and lower EQ-VAS scores; SF-36 domain scores were also substantially lower, particularly physical functioning, role-physical, bodily pain, and role-emotional (Table 2; Fig. 1). Derived health utility indices were significantly lower in PCOS/PMOS (EQ-5D-5L, 0.721 vs 0.883, *P* < .001; SF-6D, 0.669 vs 0.716, *P* < .001) (Fig. 2). Multivariable regression confirmed independent associations: PCOS/PMOS was associated with a −0.12 EQ-5D-5L utility (95% CI: −0.16 to −0.08) and a −0.04 SF-6D utility (95% CI: −0.05 to −0.02) (Tables 3 and 4). Quantile regression indicated larger deficits at lower health percentiles (Fig. 3). Older age, higher BMI, and lower income were also associated with lower utility.

**Figure 1:**
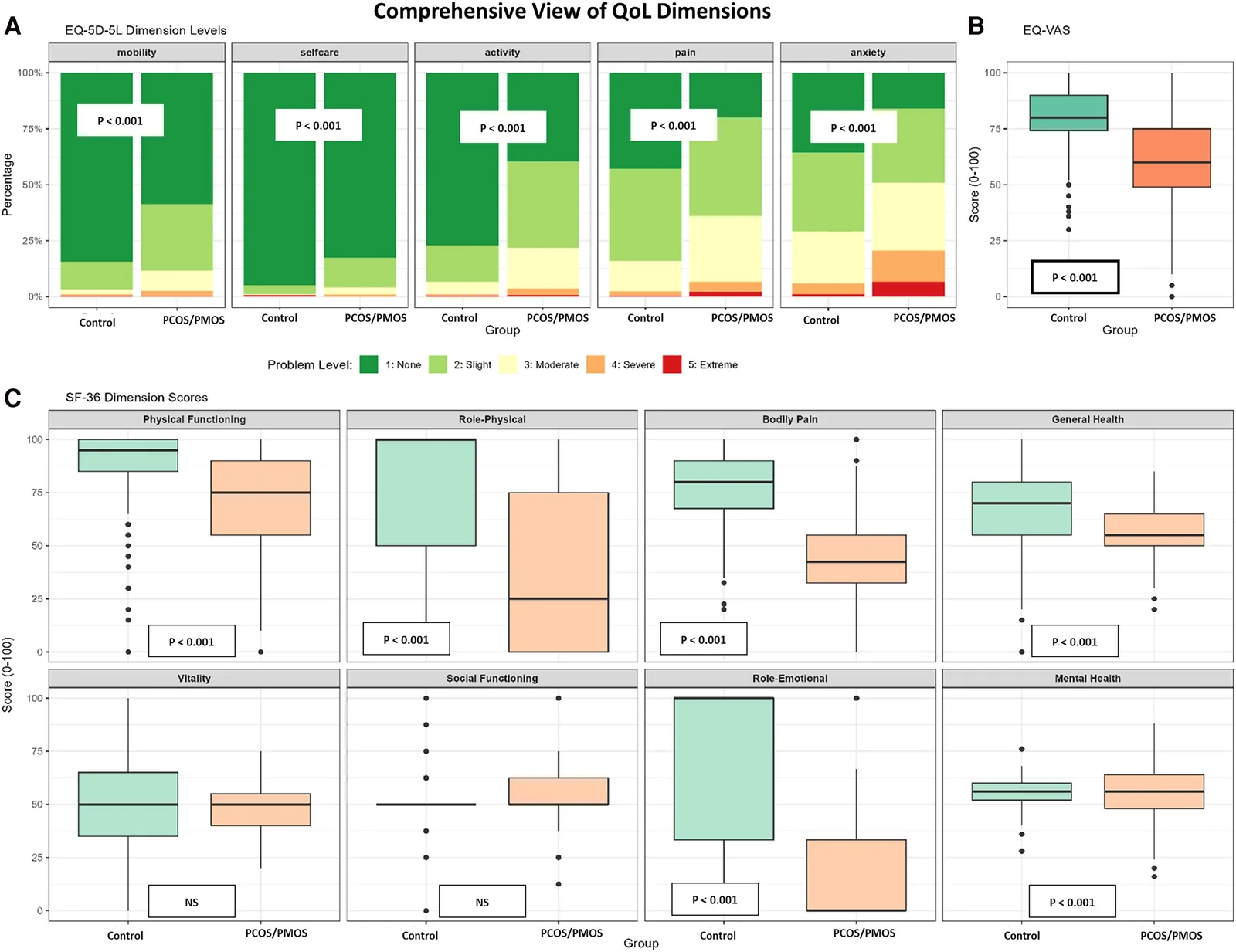
EQ-5D-5L utility index, EQ-VAS scores, and SF-36 domain scores by PCOS/PMOS status. Boxplots compare PCOS/PMOS and control groups across 3 panels. (A) EQ-5D-5L dimension levels with color-coded severity (dark green = no problem, light green = slight, yellow = moderate, orange = severe, red = extreme). (B) EQ-VAS self-rated health (0-100). (C) SF-36 domain scores (0-100). Higher scores indicate better health. Significance is shown on the figure (*P* < .001; NS, not significant; Mann–Whitney U test).

**Figure 2:**
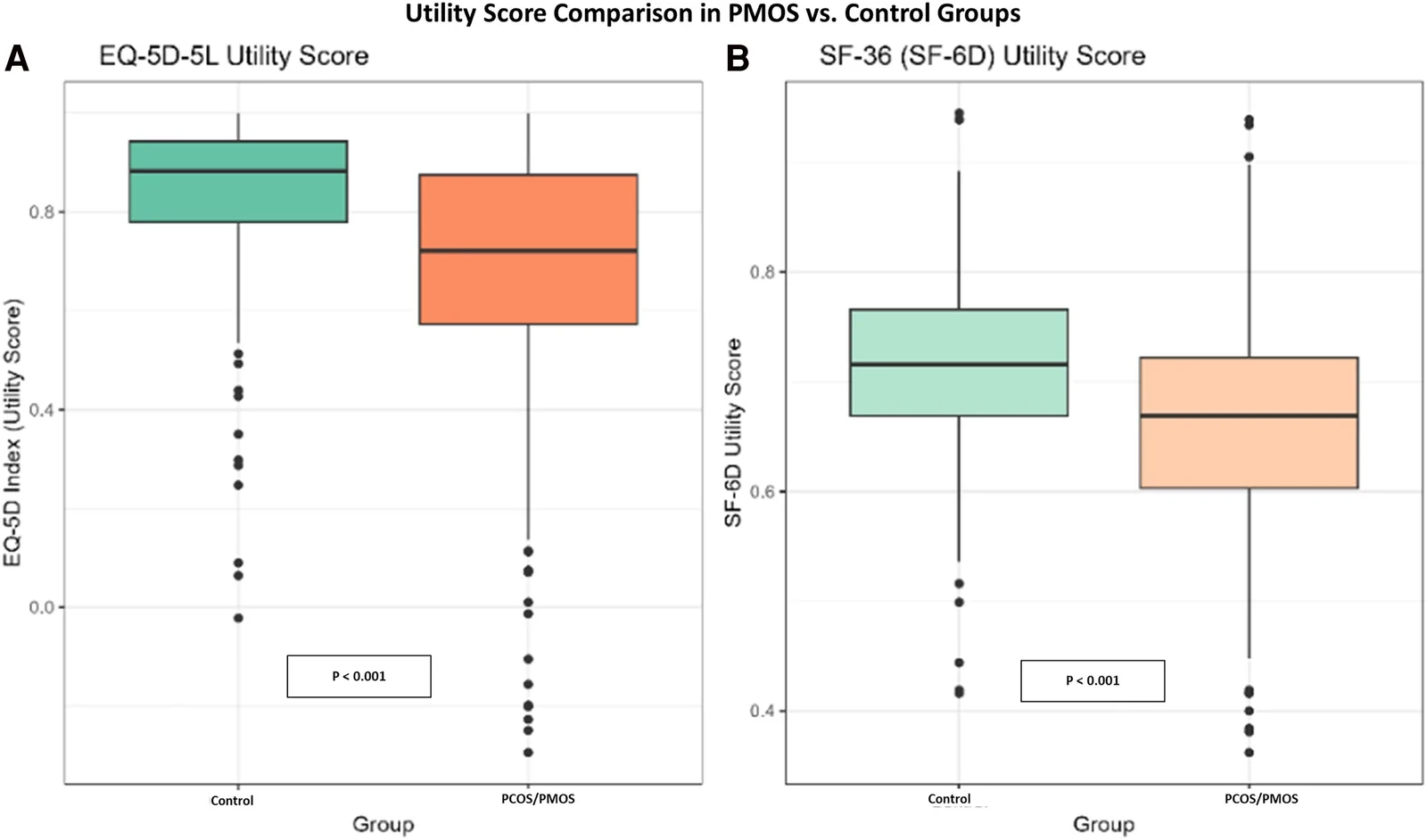
Distribution of health utility scores by PCOS/PMOS status. (A) EQ-5D-5L utility index and (B) SF-6D utility (derived from SF-36). Distribution plots with density overlays and median markers (dashed vertical lines) for utility indices (0-1 range; higher scores indicate better health). Significance is shown on the figure (all *P* < .001 from Mann–Whitney U test).

**Figure 3:**
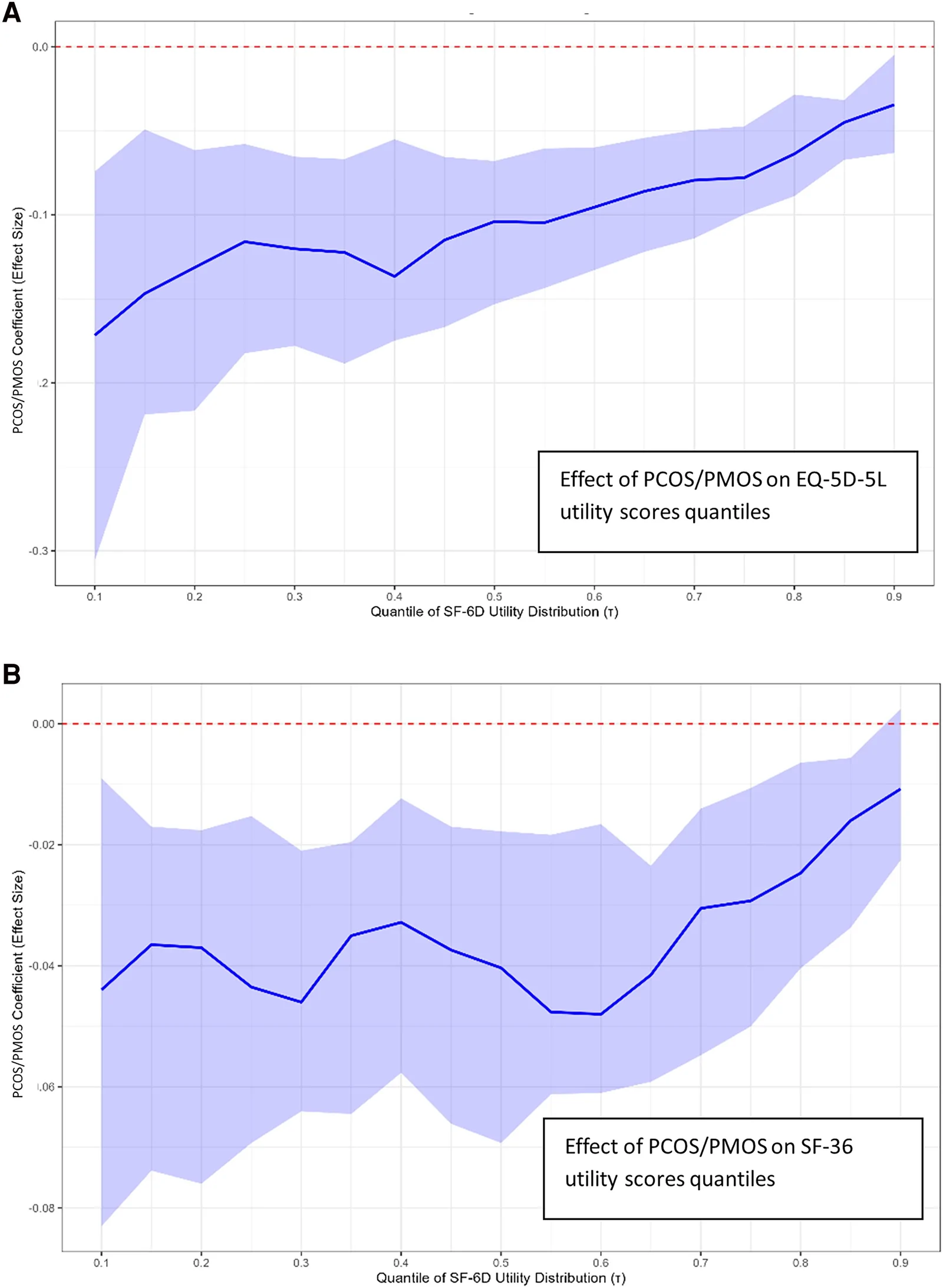
Adjusted associations of PCOS/PMOS with health utility across quantiles (quantile regression). (A) EQ-5D-5L utility index and (B) SF-6D utility. Quantile regression coefficients (β) for PCOS/PMOS across utility deciles, adjusted for age, BMI category, race, sexual orientation, education, employment, income, and marital status; shaded bands represent 95% confidence intervals.

**Table 2:** Health-related quality of life (EQ-5D-5L and SF-36), health utility scores, work productivity (WPAI-GH), and willingness to pay (WTP) among women with and without PCOS/PMOS

Variables	Response level	PCOS/PMOS (N = 433)	PCOS/PMOS controls (N = 252)	*P* value	SMD
EQ-5D-5L					
Mobility	No problem	254 (58.66)	213 (84.52)	<.001	0.530
	Slight problem	129 (29.79)	31 (12.30)		
	Moderate problem	39 (9.01)	5 (1.98)		
	Severe problem	10 (2.31)	2 (0.79)		
	Unable to walk	1 (0.23)	1 (0.40)		
Self-care	No problem	358 (82.68)	239 (94.84)	<.001	0.298
	Slight problem	57 (13.16)	10 (3.97)		
	Moderate problem	14 (3.23)	1 (0.40)		
	Severe problem	4 (0.92)	2 (0.79)		
	Unable to wash or dress	0	0		
Usual activities	No problem	171 (39.49)	194 (76.98)	<.001	0.726
	Slight problem	167 (38.57)	41 (16.27)		
	Moderate problem	79 (18.24)	14 (5.56)		
	Severe problem	13 (3.00)	2 (0.79)		
	Unable to do usual activities	3 (0.69)	1 (0.40)	<.001	0.582
Pain/discomfort	No pain or discomfort	86 (19.86)	108 (42.86)		
	Slight pain or discomfort	191 (44.11)	104 (41.27)		
	Moderate pain or discomfort	127 (29.33)	34 (13.49)		
	Severe pain or discomfort	20 (4.62)	5 (1.98)		
	Extreme pain or discomfort	9 (2.08)	1 (0.40)		
Anxiety/depression	Not anxious or depressed	69 (15.94)	90 (35.71)	<.001	0.599
	Slightly anxious or depressed	144 (33.26)	89 (35.32)		
	Moderately anxious or depressed	131 (30.25)	58 (23.02)		
	Severely anxious or depressed	60 (13.86)	12 (4.76)		
	Extremely anxious or depressed	29 (6.70)	3 (1.19)		
EQ-VAS score	Median (IQR)	60 (49, 75)	80 (73.5, 90)	<.001	1.136
EQ-5D-5L utility index	Median (IQR)	0.721 (0.573, 0.875)	0.883 (0.779, 0.943)	<.001	0.777
SF-36					
Physical Functioning	Median (IQR)	75 (55, 90)	95 (85, 100)	<.001	−0.841
Role-physical	Median (IQR)	25 (0, 75)	100 (50, 100)	<.001	−0.973
Bodily pain	Median (IQR)	42.5 (32.5, 55)	80 (67.5, 90)	<.001	−1.509
General health	Mean ± SD	56.79 ± 11.54	65.57 ± 19.43	<.001	−0.550
Vitality	Mean ± SD	47.89 ± 9.58	48.67 ± 21.56	.268	−0.047
Social functioning	Median (IQR)	50 (50, 62.5)	50 (50, 50)	.290	0.069
Role-emotional	Median (IQR)	0 (0, 33.33)	100 (33.33, 100)	<.001	−0.918
Mental Health	Mean ± SD	55.90 ± 11.83	56.05 ± 7.38	.371	−0.015
SF-6D utility (derived from SF-36)	Median (IQR)	0.669 (0.603, 0.722)	0.716 (0.668, 0.766)	<.001	−0.504
WPAI-GH					
Employment rate		84.06%	77.78%		
Absenteeism	Median (IQR)	0 (0, 13.04)	0 (0, 0)	<.001	0.500
Presenteeism	Median (IQR)	30 (10, 60)	10 (0, 30)	<.001	0.729
Work productivity loss (impairment)	Median (IQR)	39.46 (12.81, 61.25)	6 (0, 28.74)	<.001	0.820
Activity impairment	Median (IQR)	50 (20, 70)	10 (0, 30)	<.001	1.066
WTP					
	Median (IQR)	3000 (1000, 10000)	—		
	Less than $500	89 (12.9%)	—		
	$501 to $1000	67 (9.7%)	—		
	$1001 to $5000	118 (17.2%)	—		
	$5001 to $25 000	95 (13.8%)	—		
	More than $25 000	52 (7.5%)	—		
	Unsure	12 (1.7%)	—		

Values are n (%), mean ± SD, or median (IQR).

WTP percentages are based on PCOS/PMOS N = 433.

Abbreviations: SMD, standardized mean difference. See text for key to other abbreviations.

**Table 3:** Adjusted associations of PCOS/PMOS with EQ-5D-5L utility scores using ordinary least squares (OLS) and quantile regression models

	OLS Regression			Quantile (25%)			Quantile (50%)			Quantile (75%)		
Characteristic	β	95% CI	*P* value	β	95% CI	*P* value	β	95% CI	*P* value	β	95% CI	*P* value
PCOS/PMOS												
No	Ref			Ref			Ref			Ref		
Yes	−.12	−0.16, −0.08	<.001	−.12	−0.16, −0.07	<.001	−.10	−0.14, −0.07	<.001	−.08	−0.10, −0.05	<.001
Age												
18-25 years	Ref			Ref			Ref			Ref		
26-35 years	−.03	−0.09, 0.02	.2	−.02	−0.07, 0.03	.5	−.02	−0.08, 0.03	.4	−.04	−0.06, −0.01	.012
36-45 years	−.04	−0.10, 0.01	.15	−.01	−0.06, 0.04	.7	−.03	−0.08, 0.03	.4	−.05	−0.08, −0.01	.006
46-64 years	−.08	−0.15, −0.01	.027	−.07	−0.14, 0.00	.067	−.07	−0.13, 0.00	.047	−.09	−0.13, −0.05	<.001
65 years and older	−.15	−0.26, −0.03	.013	−.20	−0.38, −0.03	.020	−.13	−0.34, 0.07	.2	−.05	−0.12, 0.02	.14
BMI												
Normal	Ref			Ref			Ref			Ref		
Underweight	−.03	−0.18, 0.13	.7	−.16	−0.40, 0.08	.2	−.01	−0.52, 0.50	>.9	.03	−0.04, 0.09	.5
Overweight	−.05	−0.10, 0.01	.086	−.07	−0.11, −0.03	.002	−.03	−0.07, 0.01	.2	−.02	−0.05, 0.01	.2
Obesity	−.06	−0.11, −0.01	.017	−.11	−0.16, −0.07	<.001	−.08	−0.12, −0.03	<.001	−.04	−0.06, −0.01	.004
Race												
White	Ref			Ref			Ref			Ref		
Black	.02	−0.03, 0.06	.4	.00	−0.05, 0.05	>.9	.04	0.00, 0.08	.051	.05	0.02, 0.08	.001
Others	−.01	−0.05, 0.04	.8	−.02	−0.09, 0.05	.6	.02	−0.03, 0.06	.5	.02	0.01, 0.04	.007
Sexuality												
Straight	Ref			Ref			Ref			Ref		
Others	−.06	−0.10, −0.02	.007	−.07	−0.12, −0.01	.014	−.05	−0.09, −0.01	.019	−.05	−0.08, −0.02	.004
Education												
High school	Ref			Ref			Ref			Ref		
Bachelor	−0.02	−0.09, 0.05	.5	.01	−0.11, 0.14	.8	.02	−0.10, 0.14	.8	−.02	−0.09, 0.05	.5
Master	−.01	−0.09, 0.06	.7	0.02	−0.10, 0.14	.7	.02	−0.11, 0.14	.8	−.03	−0.09, 0.04	.5
Employment												
Yes	Ref			Ref			Ref			Ref		
No	−.08	−0.13, −0.04	<.001	−.10	−0.19, −0.01	.036	−.05	−0.11, 0.00	.041	−.04	−0.08, 0.01	.085
Income level												
Less than $10 000	Ref			Ref			Ref			Ref		
$10 000 to $49 999	.08	−0.01, 0.17	.10	.11	−0.24, 0.46	.5	.04	−0.07, 0.14	.5	.05	−0.04, 0.14	.3
$50 000 to $99 999	.14	0.05, 0.23	.003	.20	−0.15, 0.55	.3	.09	−0.01, 0.19	.075	.08	−0.01, 0.17	.071
$100 000 to $149 999	.17	0.08, 0.27	<.001	.23	−0.12, 0.58	.2	.13	0.02, 0.23	.023	.10	0.01, 0.19	.024
More than $150 000	.20	0.10, 0.30	<.001	.27	−0.08, 0.62	.13	.15	0.04, 0.25	.005	.11	0.02, 0.20	.012
Marital status												
Married	Ref			Ref			Ref			Ref		
Unmarried	.01	−0.03, 0.04	.8	.00	−0.04, 0.04	>.9	.00	−0.03, 0.03	>.9	−.02	−0.04, 0.01	.14

Coefficients are adjusted differences in mean utility scores (OLS) or differences at the 25th, 50th, and 75th percentiles (quantile regression), with 95% confidence intervals (CI) and *P* values. All models were adjusted for age group, BMI category, race, sexual orientation, education, employment, income, and marital status.

Reference categories are shown as “Ref.”

Abbreviations: β, beta regression coefficient; CI, confidence interval; OLS, ordinary least squares.

**Table 4:** Adjusted associations of PCOS/PMOS with SF-6D utility scores using ordinary least squares (OLS) and quantile regression models

	OLS Regression			Quantile (25%)			Quantile (50%)			Quantile (75%)		
Characteristic	β	95% CI	*P* value	β	95% CI	*P* value	β	95% CI	*P* value	β	95% CI	*P* value
PCOS/PMOS												
No	Ref			Ref			Ref			Ref		
Yes	−.04	−0.05, −0.02	<.001	−.04	−0.06, −0.02	<.001	−.04	−0.06, −0.02	<.001	−.03	−0.04, −0.01	<.001
Age												
18-25 years	Ref			Ref			Ref			Ref		
26-35 years	−.02	−0.04, 0.00	.084	−.04	−0.06, −0.01	.003	−.03	−0.05, −0.01	.002	−.02	−0.03, 0.00	.017
36-45 years	−.04	−0.06, −0.01	.002	−.06	−0.09, −0.03	<.001	−.04	−0.06, −0.02	<.001	−.02	−0.04, 0.00	.014
46-64 years	−.05	−0.08, −0.02	.001	−.08	−0.11, −0.04	<.001	−.05	−0.07, −0.03	<.001	−.04	−0.06, −0.01	.003
65 years and older	−.13	−0.18, −0.08	<.001	−.20	−0.26, −0.14	<.001	−.09	−0.25, 0.06	.2	−.06	−0.10, −0.01	.021
BMI												
Normal	Ref			Ref			Ref			Ref		
Underweight	.02	−0.05, 0.09	.6	.02	−0.03, 0.07	.4	.01	−0.03, 0.05	.6	.03	−0.12, 0.19	.7
Overweight	.01	−0.02, 0.03	.6	.02	−0.01, 0.04	.14	.00	−0.02, 0.02	.9	.00	−0.01, 0.01	>.9
Obesity	−.02	−0.04, 0.01	.2	−.01	−0.03, 0.02	.6	−.02	−0.05, 0.00	.048	−.02	−0.03, 0.00	.010
Race												
White	Ref			Ref			Ref			Ref		
Black	.01	−0.01, 0.03	.2	.02	−0.01, 0.05	.2	.01	−0.01, 0.02	.3	.00	−0.01, 0.01	.6
Others	.01	0.00, 0.03	.12	.00	−0.02, 0.02	.8	.00	−0.02, 0.02	.9	.02	0.00, 0.03	.007
Sexual orientation												
Straight	Ref			Ref			Ref			Ref		
Others	−.01	−0.03, 0.01	.3	−.02	−0.03, 0.00	.090	.01	−0.01, 0.03	.4	−.01	−0.02, 0.01	.3
Education												
High school	Ref			Ref			Ref			Ref		
Bachelor	−.02	−0.05, 0.00	.10	−.02	−0.07, 0.03	.5	−.03	−0.06, 0.00	.041	−.02	−0.04, 0.00	.034
Master	−.02	−0.05, 0.01	.2	−.01	−0.06, 0.05	.9	−.02	−0.05, 0.02	.3	−.01	−0.03, 0.01	.3
Employment												
Yes	Ref			Ref			Ref			Ref		
No	.00	−0.02, 0.02	.7	−.01	−0.03, 0.01	.4	.00	−0.02, 0.01	.6	.01	−0.01, 0.02	.5
Income level												
Less than $10 000	Ref			Ref			Ref			Ref		
$10 000 to $49 999	.04	0.00, 0.08	.068	.02	−0.01, 0.05	.13	.03	0.01, 0.06	.003	.05	0.02, 0.07	<.001
$50 000 to $99 999	.03	−0.01, 0.06	.2	.01	−0.02, 0.03	.6	.01	−0.01, 0.04	.2	.03	0.01, 0.05	.005
$100 000 to $149 999	.04	0.00, 0.08	.051	.03	0.00, 0.06	.025	.02	0.00, 0.05	.075	.03	0.01, 0.05	.002
More than $150 000	.05	0.01, 0.10	.018	.04	0.00, 0.07	.037	.03	0.00, 0.06	.10	.05	0.02, 0.07	<.001
Marital status												
Married	Ref			Ref			Ref			Ref		
Unmarried	−.005	−0.02, 0.01	>.9	.005	−0.01, 0.02	.6	−.005	−0.02, 0.01	.2	−.01	−0.02, 0.00	.2

Coefficients represent adjusted differences in mean SF-6D utility (OLS) or at the 25th, 50th, and 75th utility percentiles (quantile regression), with 95% confidence intervals (CI) and *P* values. All models were adjusted for age group, BMI category, race, sexual orientation, education, employment, income, and marital status.

Reference categories are shown as “Ref.”

Abbreviations: β, beta regression coefficient; CI, confidence interval; OLS, ordinary least squares.

### Indirect (work productivity-related) costs

Despite similar employment rates, women with PCOS/PMOS had significantly greater absenteeism, presenteeism, overall work impairment, and activity impairment (all *P* < .001) (Table 2; Fig. 4). The median overall work productivity loss was 39.46% in PCOS/PMOS vs 6.0% in controls. The annual indirect cost per *employed* woman was $30 368 for PCOS/PMOS and $4618 for controls. After weighting by employment rates (84.53% and 79.37%), the per-woman costs (including nonemployed) were $25 673 and $3665, respectively. The resulting excess indirect cost was $22 008 per woman/year (Table 5; Table S1) (48). Considering the number of women affected with PCOS/PMOS currently in the United States, the total yearly indirect cost in 2026 USD was estimated to be $97.9 billion.

**Figure 4:**
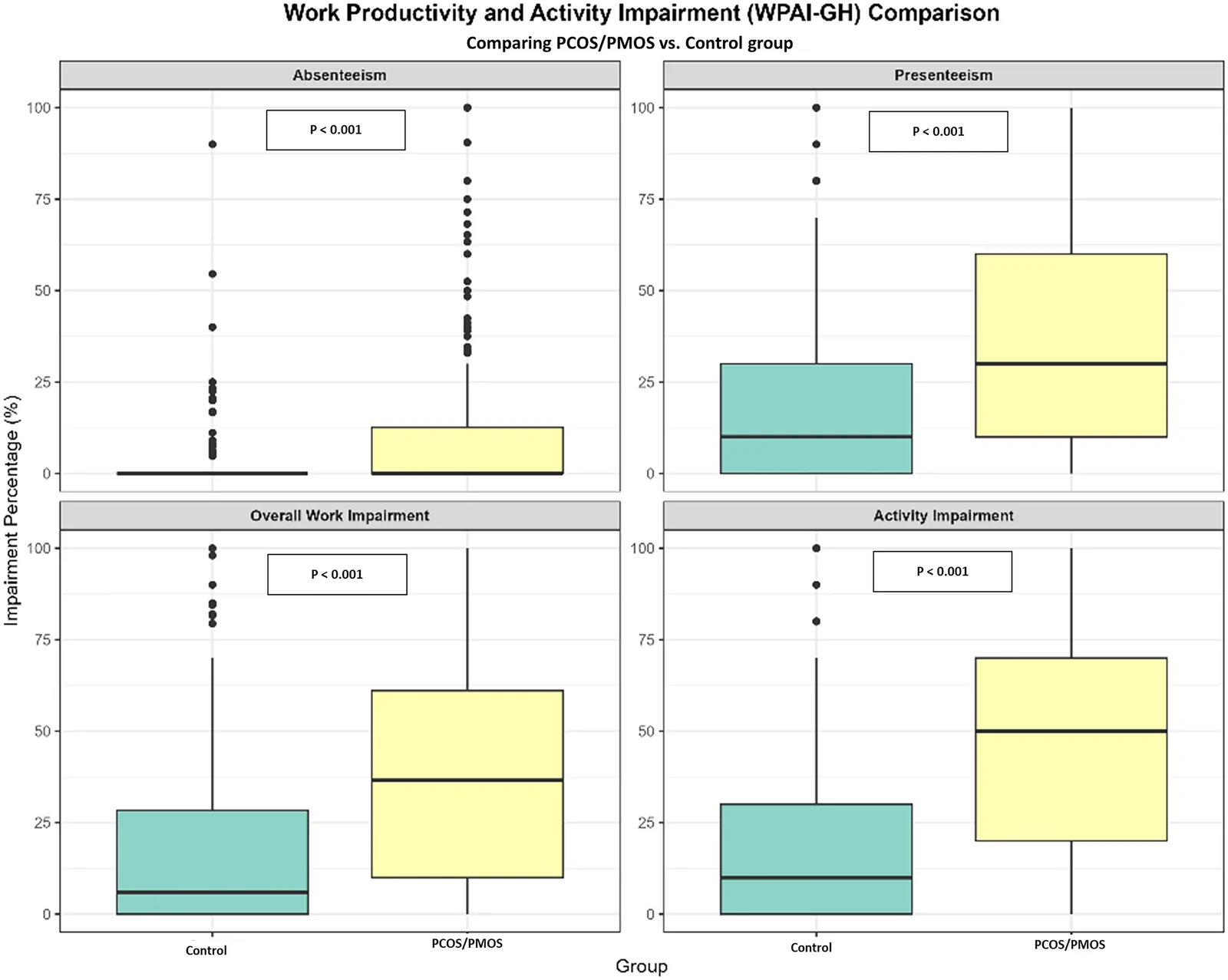
WPAI-GH outcomes by PCOS/PMOS status: absenteeism, presenteeism, overall work productivity loss, and activity impairment. Boxplots of impairment percentages (0-100%; higher percentages indicate greater impairment) across 4 WPAI-GH metrics, comparing employed PCOS/PMOS and control groups. Significance is shown on the figure (all *P* < .001 from Mann–Whitney U test).

**Table 5:** Annual excess intangible and indirect costs per woman with PCOS/PMOS

Cost component	Instrument/measure	Excess metric (95% CI)	Per woman cost at base case (95% CI)	$50 000/QALY	$150 000/QALY
Intangible	EQ-5D-5L (regression)	0.12 QALYs (0.08-0.16)	$12 000 ($8000-$16 000)*^a^*	$6000	$18 000
	SF-6D (regression)	0.04 QALYs (0.02-0.05)	$4000 ($2000-$5000)*^a^*	$2000	$6000
Indirect	WPAI-GH overall work loss	33.46 pp (median diff.)	$22 008*^b^*	—	—
Combined (EQ-5D-5L + indirect)	—	—	$34 008	—	—

All components based on 4.45 million women with PCOS/PMOS in the United States.

Values rounded to nearest $0.1 billion.

^a^Base case $100 000/QALY.

^*b*^Human capital method with employment weighting (PCOS/PMOS 84.53%, controls 79.37%), 2026 USD. No CI was reported because the estimate is derived from medians. All values rounded to nearest $0.1 billion.

### Quality-adjusted life year loss and intangible (quality of life-related) costs

The adjusted annual QALY loss was 0.12 using EQ-5D-5L and 0.04 using SF-6D (Table 5). At the base-case of $100 000/QALY, the intangible cost per woman/yr. was $12 000 (range $8000–$16 000) for EQ-5D-5L and $4000 per woman/yr. ($2000–$5000) for SF-6D. Sensitivity analyses at $50 000 and $150 000/QALY yielded $6000 to $18 000 (EQ-5D-5L). Considering the number of women affected with PCOS/PMOS currently in the United States, the total yearly intangible cost in 2026 USD was estimated by EQ-5D-5L to be $53.4 billion and $17.8 billion by the SF-6D.

### Total annual economic burden

Combining the newly estimated indirect and intangible costs with previously published direct healthcare costs yielded a total annual excess societal burden of $133.3 to $168.9 billion in 2026 USD (Table 6; Table S1) (48).

**Table 6:** Aggregate annual excess societal costs of PCOS/PMOS in the United States (2026 USD)

Cost component	Amount (billions)
Direct healthcare costs***^a^***	$17.6
Indirect costs (work productivity loss)	$97.9
Intangible costs	
Per EQ-5D-5L	$53.4
Per SF-6D	$17.8
Total	$133.3 to $168.9

^*a*^Based on previous studies (10, 13, 39, 40) updated to 2026 USD using the Medical Care Consumer Price Index (CPI) (48). The CPI ratios were 591.677/525.276 = 1.126 for 2021 to 2026, and 591.677/549.084 = 1.078 for 2023 to 2026, yielding $17.577 billion, rounded to $17.6 billion.

### Willingness to pay

The median self-reported WTP for a complete cure among women with PCOS/PMOS that subjects could realistically afford was $3000 (IQR 1000-10 000).

### Machine-learning analyses

Shapley Additive exPlanations analysis identified pain, anxiety/depression, usual activities limitations, and productivity loss as the domains most strongly associated with discriminating PCOS/PMOS from controls (Fig. 5). In SF-36 models, bodily pain, vitality, and physical functioning were most salient.

**Figure 5:**
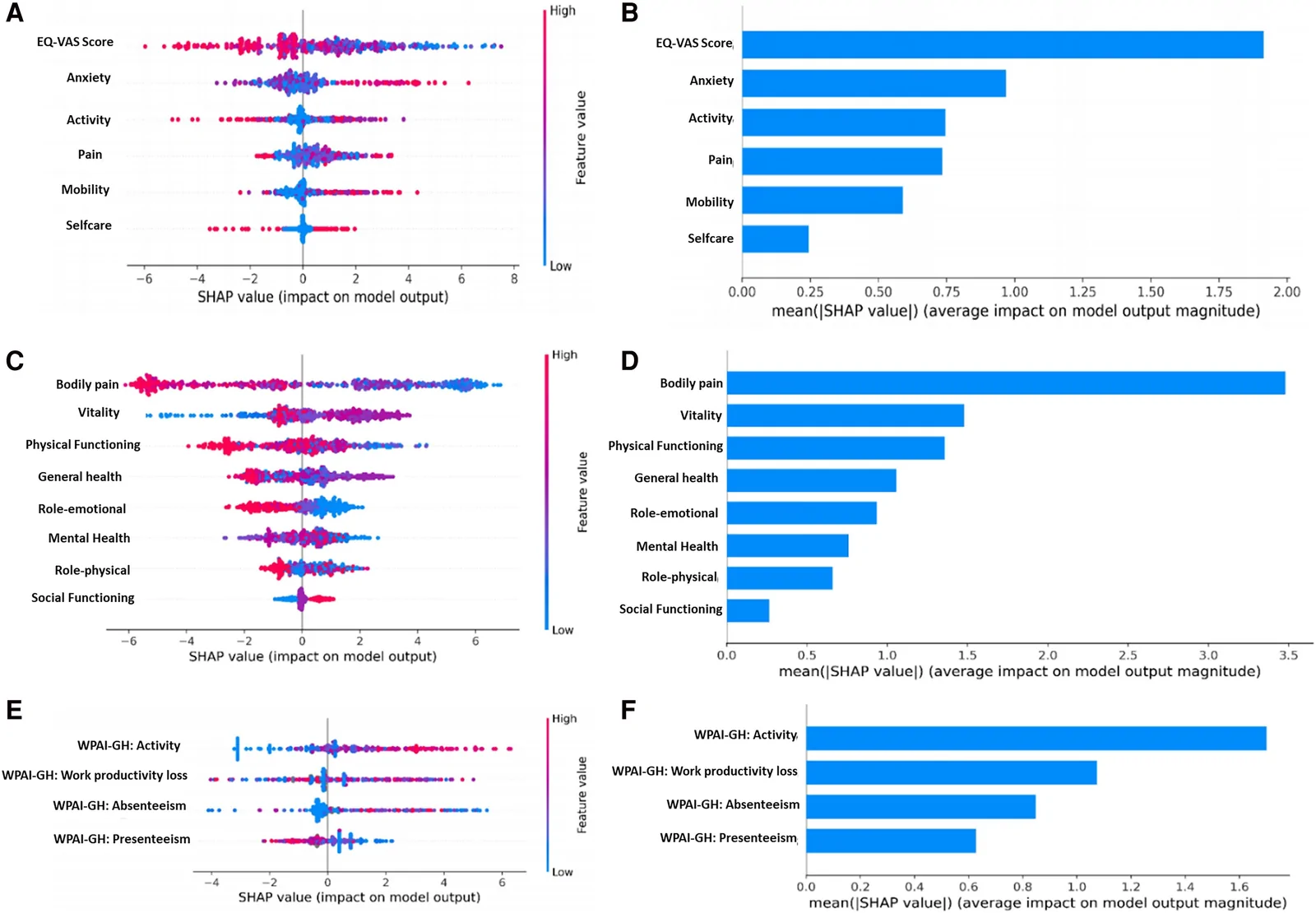
SHAP feature importance for classifying PCOS/PMOS vs controls. (A and B) EQ-5D-5L features, (C and D) SF-36 domain scores, and (E and F) WPAI-GH metrics. Beeswarm plots (A, C, and E) show SHAP value distributions for each feature; colors indicate feature values (red = high, blue = low). Bar plots (B, D, and F) rank features by mean absolute SHAP value, indicating global importance. Positive SHAP values increase PCOS/PMOS classification probability.

## Discussion

In this registry-based sample of US women, PCOS/PMOS was associated with substantial impairments in QoL, health utility, and work productivity, corresponding to an annual QALY loss of 0.12 (EQ-5D-5L) and a combined excess economic burden of approximately $34 008 per woman per year ($12 000 intangible + $22 008 indirect). Considering the number of women with PCOS/PMOS currently in the United States, the total yearly indirect (work productivity-related) cost in 2026 USD was estimated to be $97.9 billion, and the yearly intangible (QoL-related) was estimated to range from $17.8 billion (by SF-6D) to $53.4 billion (by EQ-5D-5L). These data underscore the enormous societal impact of PCOS/PMOS that extends far beyond the medical care required. The median self-reported WTP for a complete cure was $3000. The median self-reported WTP for a complete cure was $3000. Since respondents were explicitly instructed to answer within their financial means, this estimate reflects both affordability and perceived value; consequently, it should not be interpreted as a direct monetized valuation of the underlying disease burden.

Our group has previously quantifies the direct healthcare economic burden estimates of PCOS/PMOS in a series of published studies, including diagnostic and reproductive costs (menstrual dysfunction/abnormal uterine bleeding, subfertility, and hirsutism) (39), obstetrical (gestational diabetes, gestational hypertension, and pre-eclampsia) (40), mental health (anxiety, depression, and eating disorders) (13), neoplastic (endometrial cancer) (10), and increased cardiometabolic risks (T2DM and stroke) (40). In the present analysis, these previously published direct cost estimates were updated to 2026 USD and integrated with the indirect and intangible cost estimates derived from our study sample. Applying the resulting incremental cost estimates to the estimated number of US women with classic PCOS/PMOS (phenotypes A and B) (1, 50) yields a total annual excess economic burden of $133.3 to $168.9 billion (Table 6; Table S1) (48). Notably, direct medical costs represent only 10% to 13% of this, while the costs of evaluating and diagnosing affected women account for less than 1%. In contrast, indirect costs (58-73% of the total) and intangible costs (13-32% of the total) together account for the major portion of the economic burden. This distribution underscores that the main economic impact of PCOS/PMOS arises from reduced work productivity and lower health-related QoL rather than from direct medical expenditures alone.

Women with PCOS/PMOS consistently reported poorer health utility and greater functional limitations than women without PCOS/PMOS, even after adjusting for age, BMI, and sociodemographic characteristics. These differences were observed across multiple patient-reported outcome instruments and analytic approaches, indicating that the burden of PCOS/PMOS extends beyond reproductive manifestations to broader aspects of physical and psychological health. Notably, the magnitude of the association varied across the distribution of health utility scores, with larger effects among women with poorer baseline health status. These impairments in health-related QoL, spanning the physical, emotional, and social dimensions, reflect the multisystem effects of PCOS/PMOS on daily functioning (14, 32, 56). Health utility measures, such as the EQ-5D-5L and SF-6D, summarize multidimensional health states into a single value (33, 36).

Women with PCOS/PMOS demonstrated consistently lower utility and poorer QoL across physical functioning, pain, and emotional well-being, where hyperandrogenic symptoms, menstrual irregularities, metabolic disturbances, and psychological comorbidities collectively diminish daily functioning (19, 20, 27). This burden was not uniform across socioeconomic strata; lower household income was independently associated with greater utility loss (Tables 3 and 4), suggesting that the QoL impact of PCOS/PMOS may be most pronounced among those with fewer economic resources. However, the present dataset did not include detailed geographic or healthcare access measures, which could further modify this relationship and warrant attention in future studies.

Beyond perceived health status, PCOS/PMOS was linked to significant declines in health-related work productivity. Despite similar employment rates, women with PCOS/PMOS reported substantially greater absenteeism, presenteeism, and overall work impairment, indicating greater health-related burden among employed participants. These findings align with prior evidence that PCOS/PMOS negatively affects occupational functioning (19, 20) and with studies reporting that reproductive symptoms can affect work attendance and on-the-job functioning (22-25, 57-59). However, functional burden may extend across adulthood and is not limited to reproductive symptoms. Symptoms commonly experienced in PCOS/PMOS, including menstrual disturbances, pain, fatigue, sleep apnea, and psychological distress, may reduce concentration, energy levels, and physical functioning (18, 60). In addition, metabolic and mental health comorbidities frequently associated with PCOS/PMOS likely further exacerbate these limitations over time.

Our findings suggest an interrelated pattern linking symptom burden, work impairment, and psychological distress. The physical and psychological domains most affected in PCOS/PMOS, particularly pain, anxiety/depression, and limitations in usual activities, likely contribute to the elevated presenteeism and work productivity loss observed in our sample. This occupational impairment may generate financial strain and job insecurity, both established risk factors for worsening anxiety and depression. The resulting psychological distress can further amplify symptom perception and increase healthcare utilization, consistent with prior evidence that mental health disorders are a major driver of PCOS-/PMOS-attributable direct costs (13). The long-term occupational consequences of this cycle may extend well beyond what is captured by this cross-sectional assessment. Longitudinal evidence indicates that women with PCOS/PMOS have poorer work ability at midlife and higher rates of disability retirement compared to women without PCOS/PMOS (20), suggesting that the presenteeism and activity impairment in our study may represent early markers of cumulative occupational disadvantage, with lasting consequences for career trajectory and economic stability. Interrupting this cycle requires integrated care that simultaneously addresses physical symptoms, psychological health, and functional support.

The indirect excess cost of $22 008 per woman per year reflects the combined impact of absenteeism and presenteeism. The present study extends the literature by directly quantifying the QALY loss and its monetary value. A QALY loss of 0.12 (per EQ-5D-5L) translates to approximately 1.4 months of perfect health lost per year per woman. Over a 5-year period, this accumulates to more than 7 months of lost perfect health. This decrement is substantial and comparable, or even greater than, in magnitude to utility losses reported for other common chronic conditions, including diabetes and asthma in a nationally representative US sample (44), although differences in utility instruments and analytic models should be considered. Valued at the conventional US threshold of $100 000 per QALY, the intangible cost is $12 000 per woman annually (range $4000-$12 000 with SF-6D), representing a substantial aggregate burden that is not reflected in direct healthcare expenditures alone. The SF-6D estimate is lower but still meaningful, confirming that even a conservative valuation yields nontrivial societal costs.

Our machine-learning analyses provide complementary insight into which health domains most strongly differentiate women with PCOS/PMOS from controls. Pain, anxiety or depression, limitations in usual activities, and productivity loss emerged as the most discriminative features, consistent with the observed differences in QoL and work productivity. In SF-36 models, bodily pain, vitality, and physical functioning were most salient. The prominence of pain aligns with recent evidence identifying dysmenorrhea, abdominal pain, and pelvic pain as underexplored and clinically important in PCOS/PMOS (61). These findings highlight the importance of holistic management approaches that address not only reproductive and metabolic aspects but also psychological, pain-related, and functional health domains. The convergence of conventional regression and machine-learning results reinforces the robustness of our conclusions.

Several evidence-based interventions may improve QoL in PCOS/PMOS. The 2023 international guideline recommends holistic, multidisciplinary care encompassing lifestyle modification, psychological therapy, and phenotype-tailored pharmacotherapy, with lifestyle interventions tailored to patients’ needs, and benefits recognized, even in the absence of weight loss (62). Meta-analytic evidence suggests that psychological and mind–body interventions may improve anxiety and certain domains of disease-specific QoL in women with PCOS/PMOS, although the certainty of evidence remains limited (63-65). In a randomized controlled trial, an intensive lifestyle intervention designed to induce weight loss and androgen suppression via oral contraceptives independently improved multiple health-related QoL domains, with combined treatment yielding additional benefit (66). A crossover trial confirmed that oral contraceptive therapy improved QoL regardless of progestin component (67). Given that pain emerged among the most discriminative features in our machine-learning analysis, improved pain management represents an important clinical target; a recent large-scale study found that 19.21% of women with PCOS/PMOS have documented dysmenorrhea, abdominal, or pelvic pain and that treatment with oral contraceptives, metformin, or spironolactone was associated with reduced pain diagnoses (61). Whether improvements in these domains translate into measurable gains in health utility, QALYs, or work productivity remains to be established.

This study has several strengths. This is among the first studies to simultaneously evaluate QoL, health utility, and work productivity in a controlled comparison of US women with and without PCOS/PMOS and to monetize both intangible and indirect costs together with direct healthcare expenditures. The use of 2 validated preference-based instruments (EQ-5D-5L and SF-6D) enabled standardized estimation of health utility values and QALY losses, enhancing comparability with other disease areas. Additionally, the analytic framework combined conventional multivariable regression, quantile regression, and machine-learning approaches, providing both adjusted effect estimates and exploration of heterogeneity across the health distribution.

Several limitations should be noted. The cross-sectional design precludes causal inference, and PCOS/PMOS status was self-reported rather than clinically verified, which may introduce misclassification. Recruitment through an online registry and underrepresentation of older women may limit generalizability to the broader PCOS/PMOS population, particularly later adulthood. Detailed clinical phenotyping, disease severity, treatment information, and age at diagnosis were not available; although duration since diagnosis was captured in broad categories, this does not permit estimation of age at diagnosis or assessment of its potential influence on QoL and economic outcomes. Geographic data were limited to 3-digit ZIP codes, precluding rural–urban classification, and healthcare access was not directly measured. These factors may influence the burden estimates and warrant further investigation. Additionally, the economic estimates reflect a point-in-time assessment in 2026 USD and do not account for future changes in healthcare costs, treatment patterns, or disease prevalence. Consistent with prior PCOS/PMOS economic burden studies, national aggregate estimates were based on the US female population aged 15-44 years and a prevalence estimate for classic PCOS/PMOS phenotypes A and B. Accordingly, the estimates do not capture potential burden outside this modeled population. Trajectory modeling would require longitudinal data with repeated measures of health utility, work productivity, and healthcare expenditures. Despite these limitations, the consistent results across multiple validated instruments and analytic methods support the internal validity of our findings.

In conclusion, PCOS/PMOS is associated with meaningful reductions in health utility, measurable QALY losses, significant health-related impairments in work productivity, and a substantial excess economic burden. The total annual societal burden of PCOS/PMOS ranges from nearly $133 to $169 billion, predominantly the result of indirect and intangible costs. This underscores the profound economic impact of the syndrome and reinforces the need for multidisciplinary management and support strategies that address not only reproductive and metabolic outcomes but also pain, mental health, physical functioning, and daily activity limitations. Interpreted in this context, these findings highlight the importance of future studies evaluating whether targeted clinical, payer, workplace, and broader policy approaches can reduce contributors to absenteeism, presenteeism, indirect costs, and intangible burden. Overall, PCOS/PMOS is a multisystem condition with meaningful functional, physical, and societal consequences, often compounded by delayed diagnosis and gaps in comprehensive care (62, 68).

## Data Availability

The data that support the findings of this study are available from the corresponding author upon reasonable request.
